# Author Correction: Vascular surveillance by haptotactic blood platelets in inflammation and infection

**DOI:** 10.1038/s41467-022-31310-7

**Published:** 2022-08-08

**Authors:** Leo Nicolai, Karin Schiefelbein, Silvia Lipsky, Alexander Leunig, Marie Hoffknecht, Kami Pekayvaz, Ben Raude, Charlotte Marx, Andreas Ehrlich, Joachim Pircher, Zhe Zhang, Inas Saleh, Anna-Kristina Marel, Achim Löf, Tobias Petzold, Michael Lorenz, Konstantin Stark, Robert Pick, Gerhild Rosenberger, Ludwig Weckbach, Bernd Uhl, Sheng Xia, Christoph Andreas Reichel, Barbara Walzog, Christian Schulz, Vanessa Zheden, Markus Bender, Rong Li, Steffen Massberg, Florian Gaertner

**Affiliations:** 1 Medizinische Klinik und Poliklinik I, Klinikum der Ludwig-Maximilians-Universität, 81377 Munich, Germany; 2 DZHK (German Centre for Cardiovascular Research), Partner Site Munich Heart Alliance, 80802 Munich, Germany; 3 Ludwig-Maximilians-Universität, 80799 Munich, Germany; 4 Walter-Brendel-Centre of Experimental Medicine, University Hospital, München, Germany; 5 Institute of Cardiovascular Physiology and Pathophysiology, Biomedical Center, Planegg-Martinsried, Munich, Germany; 6 Department of Otorhinolarynology, Ludwig-Maximilians-Universität Munich, Munich, Germany; 7 Department of Cell Biology, Johns Hopkins University School of Medicine, 855 North Wolfe Street, Baltimore, MD 21205 USA; 8 Institute of Science and Technology (IST) Austria, 3400 Klosterneuburg, Austria; 9 Institute of Experimental Biomedicine I, University Hospital and Rudolf Virchow Center, Würzburg, Germany

**Keywords:** Cell migration, Actin, Acute inflammation, Platelets

Correction to: *Nature Communications* 10.1038/s41467-020-19515-0, published online 13 November 2020.

The original version of this Article contained an error in the “Introduction”, as reference was missing. The original version read incorrectly “[…] since the abrogation of lamellipodia formation does not affect hemostatic or thrombotic platelet functions ^22,23^ […]”. The correct version states “[…] since the abrogation of lamellipodia formation does not affect hemostatic or thrombotic platelet functions ^22,23,24^…” Ref. 24 is: ‚Schurr, Y. et al. Platelet lamellipodium formation is not required for thrombus formation and stability. Blood 134, 2318–2329 (2019). All references have been renumbered accordingly.

The original version of this Article contained an error in “Results”, as panel 2g was referenced instead of panel 2i in one instance. The original version read incorrectly “[…] triggering isotropic contraction of the matrix (Supplementary Fig. 2d–g and Supplementary Movie 2)”. The correct version states “[…] triggering isotropic contraction of the matrix (Supplementary Fig. 2d–i and Supplementary Movie 2)”.

The original version of this Article contained an error in “Results”, as figure 5 was referenced instead of figure 4 in one instance. The original version read incorrectly “[…]*Arpc2*-deficient platelets were less efficiently recruited to spots of leukocyte transmigration (Fig. 5e, f) […].” The correct version states “*Arpc2*-deficient platelets were less efficiently recruited to spots of leukocyte transmigration (Fig. 4e, f) […].”

The original version of this Article contained an error in “Results”, as panel 5h was referenced instead of panel 5i in one instance. The original version read incorrectly “[…] despite comparable platelet numbers and leukocyte recruitment patterns (Supplementary Fig. 5f–h).” The correct version states “[…] despite comparable platelet numbers and leukocyte recruitment patterns (Supplementary Fig. 5f–i).”

The original version of this Article contained an error in “Results”, as panel 5i was referenced instead of panel 5j in one instance. The original version read incorrectly “ […] treating mice with integrin α_IIb_ inhibitor tirofiban resulted in comparable microbleeds (Supplementary Fig. 5i) […]”. The correct version states “[…] treating mice with integrin α_IIb_ inhibitor tirofiban resulted in comparable microbleeds (Supplementary Fig. 5j) […]”.

The original version of this Article contained an error in “Results”, as panel 5j was referenced instead of panel 5k in one instance. The original version read incorrectly “[…] highlighting a general role of platelet migration in the prevention of microbleed independent of stimulus (Supplementary Fig. 5j).” The correct version states “[…] highlighting a general role of platelet migration in the prevention of microbleed independent of stimulus (Supplementary Fig. 5k).”

The original version of this Article contained an error in “Results”, as panel 7c was referenced instead of panel 7d in one instance. The original version read incorrectly “Next, we inoculated mice with MRSA to assess platelet–bacteria interactions in Gram-positive pneumonia (Fig. 7c and Supplementary Methods).” The correct version states “Next, we inoculated mice with MRSA to assess platelet–bacteria interactions in Gram-positive pneumonia (Fig. 7d and Supplementary Methods).”

The original version of this Article contained an error in “Results”, as figure 6 was referenced instead of figure 7 in one instance. The original version read incorrectly “[…] a fraction of bacteria disseminated in the lung vasculature (Supplementary Fig. 6f–g).” […] a fraction of bacteria disseminated in the lung vasculature (Supplementary Fig. 7f–g).”

The original version of this Article contained an error in “Results”, as panels 7c, d were referenced instead of panels 7d-f in one instance. The original version read incorrectly “[…] lamellipodia and bundled bacteria that had escaped to non-abscessing lung tissue (Fig. 7c, d and Supplementary Fig. 7g).” The correct version states “[…] lamellipodia and bundled bacteria that had escaped to non-abscessing lung tissue (Fig. 7d-f and Supplementary Fig. 7g).”

The original version of this Article contained an error in “Results”, as panels 7d, e were referenced instead of panels 7e, f in one instance. The original version read incorrectly “[…] indicating an important role for platelet spreading and migration in this process (Fig. 7d, e).” The correct version states “[…] indicating an important role for platelet spreading and migration in this process (Fig. 7e, f).”

The original version of this Article contained an error in “Results”, as panels 7f–h were referenced instead of panels 7g–i in one instance. The original version read incorrectly “[…] increased fraction of mice with disseminated MRSA in the kidney and spleen (Fig. 7f–h and Supplementary Fig. 7l).” The correct version states “[…] increased fraction of mice with disseminated MRSA in the kidney and spleen (Fig. 7g–i and Supplementary Fig. 7l).”

The original version of this Article contained an error in the “Discussion”, as reference (24) was missing. The original version read incorrectly “Yet, its physiological function remained largely enigmatic^22,23^.” The correct version states “Yet, its physiological function remained largely enigmatic^22,23,24^.”

The original version of this Article contained an error in the “Discussion”, as reference (24) was not discussed appropriately. Therefore, the following part was added: “In contrast to WASp-/-, WAVE deletion (Cyfip1-/-) abrogated large circular lamellipodia in platelets^24^. However, the short Arp2/3-dependent lamellipodial-like extensions forming at the center of Cyfip1-/- platelets, were still sufficient to promote platelet migration and haptotaxis, indicating a partial redundancy of NPFs in platelet motility.”

The original version of this Article contained an error in Fig. 4e: In the original Figure 4e the graph showing the platelet distance from endothelial junctions indicated Arpc3 +/+ and Arpc3 -/- at the x-axis. The correct version of Figure 4 is:
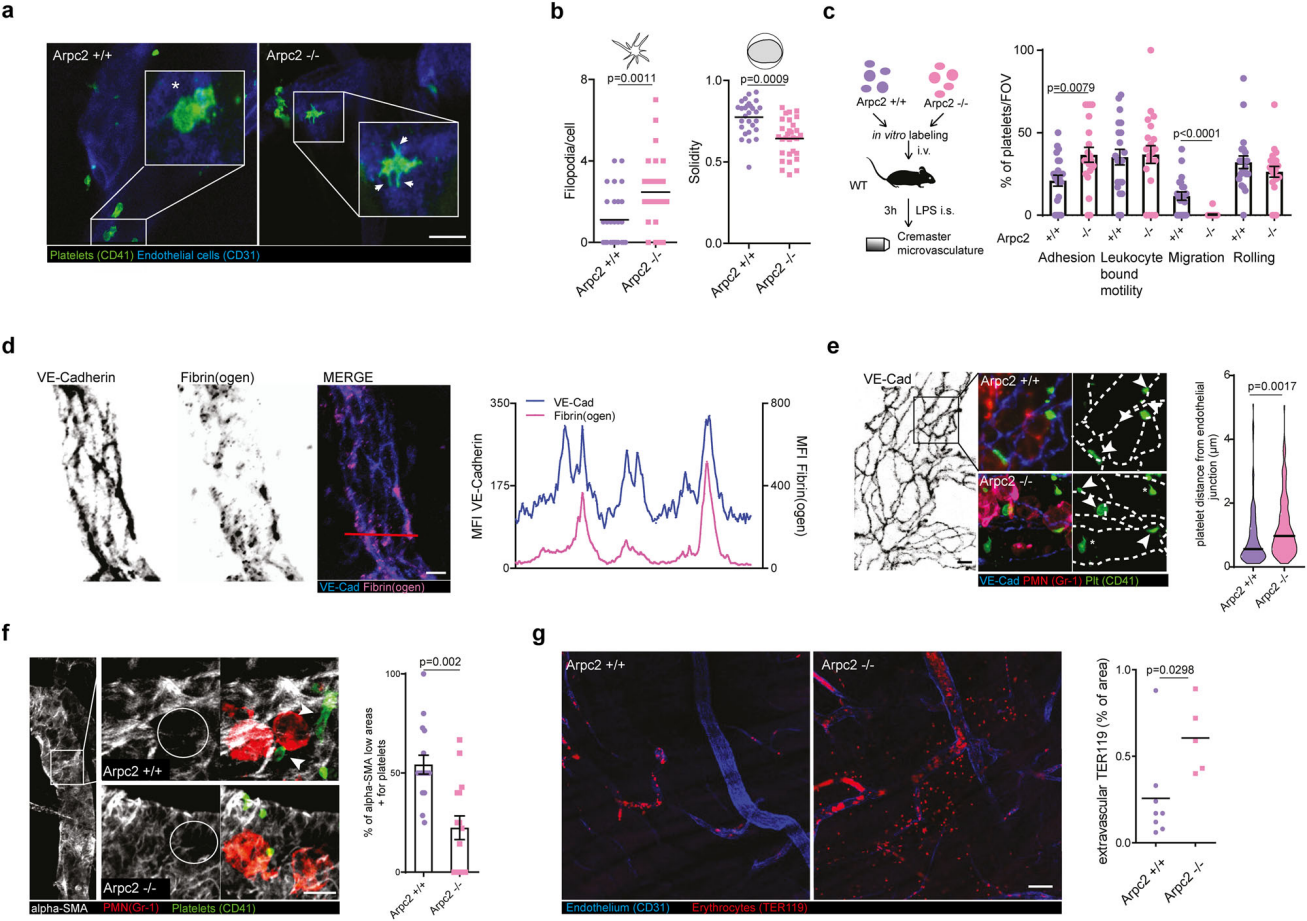


Which replaces the previous incorrect version:
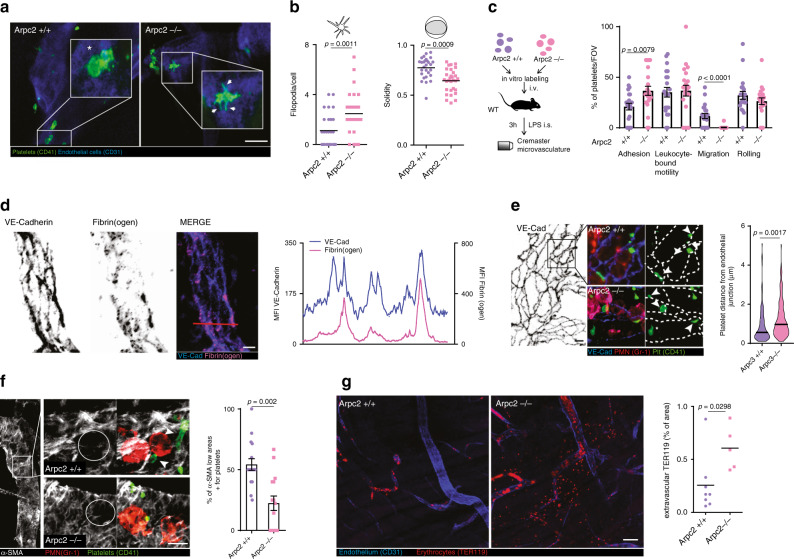


The original version of this Article contained an error in the legend of Fig. 6, and the description of panels e-g was updated, and panel e was not appropriately described. The original version read incorrectly “ […] f–g Migration of WT, *Cyfip1−/−*, and *Arpc2−/−* platelets, f representative micrographs, g percentage of cells migrating and area cleared per cell, ANOVA and Tukey’s post hoc test, n = 3 mice per group. Scale bar = 5 μm. ” The correct version states: “[…] e–g Migration of WT, *Cyfip1−/−*, and *Arpc2−/−* platelets, e representative micrographs, f percentage of cells migrating and g area cleared per cell, ANOVA and Tukey’s post hoc test, n = 3 mice per group. Scale bar = 5 μm. …”

The original version of this Article contained an error in “Acknowledgements”, as it was incomplete. The original version read incorrectly “We thank Sebastian Helmer, Nicole Blount, Christine Mann, and Beate Jantz for technical assistance; Hellen Ishikawa-Ankerhold for help and advice; Michael Sixt for critical discussions. This study was supported by the DFG SFB 914 (S.M. [B02 and Z01], K.Sch. [B02], B.W. [A02 and Z03], C.A.R. [B03], C.S. [A10], J.P. [Gerok position]), the DFG SFB 1123 (S.M. [B06]), the DFG FOR 2033 (S.M. and F.G.), […]. The correct version states: “We thank Sebastian Helmer, Nicole Blount, Christine Mann and Beate Jantz for technical assistance; Hellen Ishikawa-Ankerhold for help and advice; Laura Machesky for providing *PF4-Cre;Cyfip1*^*fl/fl*^ mice; Michael Sixt for critical discussions and the electron microscopy facility of IST Austria for excellent support. This study was supported by the DFG SFB 914 (S.M. [B02 and Z01], K.S. [B02], B.W. [A02 and Z03], C.A.R. [B03], C.S. [A10], J.P. [Gerok position]), the DFG SFB 1123 (S.M. [B06]), the DFG FOR 2033 (S.M. and F.G.), the DFG (M.B. [BE5084/3-2, TR240 project number 374031971]), […].

The source data of Supplementary Figure 1a was missing in the original version of the manuscript and has now been included in the source data file. The source data of Supplementary Figure 4a was originally labeled Arpc3+/+ Bu and Arpc3-/-. The corrected version replaces the labels with “Arpc2+/+ Bu and Arpc2-/-”, respectively. The source data of Supplementary Figure 6b was originally labeled Arpc3-/-. The corrected version replaces the labels with “Arpc2-/-”. The source data of Figure 7g was originally labeled Arpc3+/+ and Arpc3-/-. The corrected version replaces the labels with “Arpc2+/+ and Arpc2-/-”.

These errors have been corrected in the HTML and PDF versions of the article.

