# Supplementary material for: Vascular surveillance by haptotactic blood platelets in inflammation and infection
Source: Nat Commun. 2020 Nov 13;11:5778. doi: 10.1038/s41467-020-19515-0 (PMC7666582; doi:10.1038/s41467-020-19515-0)
Supplement: Supplementary file 3 — Descriptions of Additional Supplementary Files [file 41467_2020_19515_MOESM3_ESM.pdf]

Descriptions of Additional Supplementary Files

Supplementary Movie 1

Description: Platelet motility patterns and encountered microenvironment in inflammation and thrombosis in vivo. Related to Figure 1 and Supplementary Figure 1. Platelet motility patterns are shown in the inflamed (LPS) cremaster muscle using spinning disc intravital microscopy. Thrombus retraction following vascular injury with ferric chloride is shown in the mesenteric microvasculature using confocal intravital microscopy. 3D reconstruction of confocal slices of the inflamed cremaster vasculature show thin fibrinogen deposition at the vascular wall. For all movie sequences time stamps show seconds.

Supplementary Movie 2

Description: Platelets adapt their behavior to the microenvironment in vitro. Related to Figure 2 and Supplementary Figure 2. a, Platelets polarize adopting a halfmoon-like shape and migrate on Fibrinogen coated surfaces. During migration platelets remove immobilized Fibrinogen (green: Alexa-488-labeled Fibrinogen). b, Platelets entangled in cross-linked fibrin matrices radially align the fibrin meshwork, thereby triggering isotropic contraction of the matrix (green: Alexa-labeled Fibrin). c, Differential interference microscopy of platelets migrating on Fibrinogen (left) and platelets adhering to Fibrin (right). Last frame shows fluorescence image of the same field of view after 30 min (green: Alexa-488), black areas are regions where platelets have removed the ligands (Fibrinogen or Fibrin) from the substrate (coverslip). d, Platelets polarize and migrate in response to cleavage of Fibrin (green: Alexa-488) by Plasmin. Note the increased background fluorescence (green) as digested Fibrin-Alexa-488 is released into the supernatant. e, Integrin-ligands of tunable mechanical stability. left: stable: Platelets remain stationary when adhering to RGD (Arg-Gly-Asp)-peptides covalently bound (stable) to a PLL(Poly-L-lysine)-PEG(Poly-ethylene-glycol) backbone immobilized on a glass coverslip, fragile: Platelets migrate when adhering to RGD-biotin bound to a PLL-PEG-biotin backbone via a Neutravidin-FITC (NA) -bridge, that is ruptured by platelet pulling forces. For movies a-d. In movie e time stamps show minutes.

Supplementary Movie 3

Description: Platelet polarization and actin polymerization in vitro. Related to Figure 3 and Supplementary Figure 3. a, Filopodia formation of adhering platelets was followed by lamellipodia formation pointing to the direction of migration. b, Visualization of actin polymerization using platelets isolated from LifeAct-eGFP mice. Migrating platelets show actin-dense waves at the leading-edge propagating from one side to the other. Lamellipodia-formation was blocked by Arp2/3-inhibitor CK666. For all movie sequences time stamps show seconds.

Supplementary Movie 4

Description: Effect of Arp2/3 inhibition and Myosin II inhibition on platelet migration and retraction. Related to Figure 3 and Supplementary Figure 3. Platelet response to Arp2/3-inhibition (CK666) and MyosinIIa-inhibition (Blebbistatin) was visualized with phase contrast (right) and epifluorescence

microscopy (left). Fluorescence images show Alexa-488-labeled Fibrin(ogen). For all movie sequences time stamps show seconds.

Supplementary Movie 5

Description: Arpc2 -/- platelets do not migrate but retract in vivo. Related to Figure 4 and Supplementary Figure 4. Platelet motility patterns are shown in the inflamed (LPS) cremaster muscle using spinning disc intravital microscopy. Arpc2-/- (blue) and Arpc2+/+ (green) platelets are adoptively transferred into wildtype recipient mice. Thrombus retraction following vascular injury with ferric chloride is shown in the mesenteric microvasculature using confocal intravital microscopy of Arpc2-/- mice (green=platelets). For all movie sequences time stamps show seconds.

Supplementary Movie 6

Description: Cyfip1 -/- retain their ability to migrate. Related to Figure 6. Platelet migration was visualized with phase-contrast and epifluorescence microscopy (green: Alexa-488-labeled Fibrinogen). Note, in contrast to Arpc2-/- platelets, Cyfip1-/- platelets retain the ability to form premature lamellipodia-like protrusions and migrate. For all movie sequences time stamps show seconds.

Supplementary Movie 7

Description: Platelet-mediated biofilm clearance depends on Arp2/3 Related to Figure 7 and Supplementary Figure 7. Platelet-bacteria (MRSA)-interaction is visualized by time-lapse phase-contrast microscopy in the absence and presence of Arp2/3-inhibition (CK666). Confocal stacks of platelets (magenta) interacting with bacteria (green) were 3D rendered.

Supplementary Data 1

Description: Overview of performed mouse experiments

Supplementary Data 2

Description: Primers for genotyping of mouse strains
