# Supplementary material for: Vascular surveillance by haptotactic blood platelets in inflammation and infection
Source: Nat Commun. 2020 Nov 13;11:5778. doi: 10.1038/s41467-020-19515-0 (PMC7666582; doi:10.1038/s41467-020-19515-0)
Supplement: Supplementary file 13 — Reporting Summary [file 41467_2020_19515_MOESM13_ESM.pdf]

nature research

Corresponding author(s): Florian Gaertner (florian.gaertner@ist.ac.at); Steffen Massberg (steffen.massberg@med.uni-muenchen.de)

Last updated by author(s): Aug 31, 2020

Reporting Summary

Nature Research wishes to improve the reproducibility of the work that we publish. This form provides structure for consistency and transparency in reporting. For further information on Nature Research policies, see Authors & Referees and the Editorial Policy Checklist .

Statistics

For all statistical analyses, confirm that the following items are present in the figure legend, table legend, main text, or Methods section.

n/a	Confirmed	
☐	☒	The exact sample size (n) for each experimental group/condition, given as a discrete number and unit of measurement
☐	☒	A statement on whether measurements were taken from distinct samples or whether the same sample was measured repeatedly
☐	☒	The statistical test(s) used AND whether they are one- or two-sided<br>*Only common tests should be described solely by name; describe more complex techniques in the Methods section.*
☒	☐	A description of all covariates tested
☐	☒	A description of any assumptions or corrections, such as tests of normality and adjustment for multiple comparisons
☐	☒	A full description of the statistical parameters including central tendency (e.g. means) or other basic estimates (e.g. regression coefficient) AND variation (e.g. standard deviation) or associated estimates of uncertainty (e.g. confidence intervals)
☐	☒	For null hypothesis testing, the test statistic (e.g. F, t, r) with confidence intervals, effect sizes, degrees of freedom and P value noted<br>*Give P values as exact values whenever suitable.*
☒	☐	For Bayesian analysis, information on the choice of priors and Markov chain Monte Carlo settings
☒	☐	For hierarchical and complex designs, identification of the appropriate level for tests and full reporting of outcomes
☒	☐	Estimates of effect sizes (e.g. Cohen's d, Pearson's r), indicating how they were calculated

Our web collection on statistics for biologists contains articles on many of the points above.

Software and code

Policy information about availability of computer code

Data collection	cellSens 1.1.8 (Olympus)<br>ZenBlack v2 (Zeiss)<br>AxioVision v4.9 (Zeiss)<br>slideBook v6 (3i)<br>Asylum AFM software v14 (Oxford Instruments)<br>Aggrolink (Chrono Log) v7
Data analysis	FIJI (Image J 2.0.0-rc-69/152n)(used Plugins: ImageStabilizer, Manual Tracking, Chemotaxis Tool (IBIDI), Image Calculator, MosaicIA, Coloc2)<br>CellTool suite 1.0 (Zach Pincus)<br>Imaris 5.1 (BitPlane)<br>ZenBlack v2 (Zeiss)<br>Excel 365 (Microsoft)<br>IGOR Pro 7 (WaveMetrics)<br>Prism 6.0 (GraphPad)<br>Illustrator (Adobe) CS6

For manuscripts utilizing custom algorithms or software that are central to the research but not yet described in published literature, software must be made available to editors/reviewers. We strongly encourage code deposition in a community repository (e.g. GitHub). See the Nature Research guidelines for submitting code & software for further information.

Data

Policy information about availability of data

All manuscripts must include a data availability statement. This statement should provide the following information, where applicable:

- Accession codes, unique identifiers, or web links for publicly available datasets
- A list of figures that have associated raw data
- A description of any restrictions on data availability

Data that supports the findings of this study are available within the article and its Supplementary Information. Source Data for all Figures and Supplementary Figures are provided with the paper. Any additional information and related data are available upon reasonable request.

Field-specific reporting

Please select the one below that is the best fit for your research. If you are not sure, read the appropriate sections before making your selection.

[x] Life sciences   [ ] Behavioural & social sciences   [ ] Ecological, evolutionary & environmental sciences

For a reference copy of the document with all sections, see nature.com/documents/nr-reporting-summary-flat.pdf

Life sciences study design

All studies must disclose on these points even when the disclosure is negative.

Sample size	For some in vivo experiments (Tail vein bleeding experiments, acute lung injury experiments, inflammation of cremaster muscle), power analysis was performed as described in (http://www.stat.uiowa.edu/~rlenth/Power/) (Lenth, R. V. (2001), “Some Practical Guidelines for Effective Sample Size Determination,” The American Statistician, 55, 187-193.). For all other experiments established experimental group sizes from previous work were used to approximate sample sizes (Massberg et al. Nature Medicine;16:887–896(2010); Gaertner et al. Cell;171 (6):1368-1382(2017)).
Data exclusions	No data was excluded from analysis.
Replication	Replication of all data was successful. All data were replicated at least 3 times, see figure legends and source data for recapitulations of experiments.
Randomization	No randomization was performed; all cells or mice assigned to control or experimental group were analyzed.
Blinding	Investigators were not blinded to group allocation for majority of the study as data collection and analysis was performed by the same individual assigning the groups; however, the researcher who performed quantification of movement patterns of adoptively transfered Arpc2 +/+ and Arpc2 -/- platelets within the inflamed cremaster microvasculature was blinded for the color assignment to control (Arpc2+/+) and experimental (Arpc2-/-) group.

Reporting for specific materials, systems and methods

We require information from authors about some types of materials, experimental systems and methods used in many studies. Here, indicate whether each material, system or method listed is relevant to your study. If you are not sure if a list item applies to your research, read the appropriate section before selecting a response.

Materials & experimental systems

n/a	Involved in the study	
[ ]	[x]	Antibodies
[x]	[ ]	Eukaryotic cell lines
[x]	[ ]	Palaeontology
[ ]	[x]	Animals and other organisms
[ ]	[x]	Human research participants
[x]	[ ]	Clinical data

Methods

n/a	Involved in the study	
[x]	[ ]	ChIP-seq
[ ]	[x]	Flow cytometry
[x]	[ ]	MRI-based neuroimaging

Antibodies

Antibodies used	anti-mouse<br>CD45 (clone 30F-11), Biolegend, Cat. No. 103105 dilution 1:100, validation: https://www.biolegend.com/en-us/products/pe-anti-mouse-cd45-antibody-100<br>citH3 (polyclonal), abcam, Cat. No. ab5103 dilution 1:100, validation: https://www.abcam.com/histone-h3-citrulline-r2--r8--r17-antibody-ab5103.html<br>Ly6G/C (clone RB6-8C5), Biolegend, Cat. No. 108417 dilution 1:100, validation: https://www.biolegend.com/en-us/products/

alexa-fluor-488-anti-mouse-ly-6g-ly-6c-gr-1-antibody-2711
CD31 (clone MEC13.3), Biolegend, Cat. No. 102416 dilution 1:100, validation: https://www.biolegend.com/en-us/products/alexa-fluor-647-anti-mouse-cd31-antibody-3094
Ly-76(clone TER119), Biolegend, Cat. No. 116208 dilution 1:100, validation: https://www.biolegend.com/en-us/products/pe-anti-mouse-ter-119-erythroid-cells-antibody-1867
VE-Cadherin (VECD-1), Biolegend, Cat. No. 138005 dilution 1:100, validation: https://www.biolegend.com/en-us/products/purified-anti-mouse-cd144-ve-cadherin-antibody-6564
CD62P (clone RMP-1), Biolegend, Cat. No. 148303 dilution 1:100, validation: https://www.biolegend.com/en-us/products/apc-anti-mouse-rat-cd62p-p-selectin-antibody-10805x4
activated CD41 (clone JonA), Emfret, Cat. No. M023-2 dilution 1:100, validation: https://www.emfret.com/uploads/tx_beproducts/M023-2_JON_A.pdf

GpIb (DyLight488-labelled, derivatized, polyclonal, Emfret), Cat. No. X488 dilution 1:100, validation: https://www.emfret.com/fileadmin/user_upload/Datasheets/X488.pdfx
GpIb (DyLight649-labelled, derivatized, polyclonal, Emfret), Cat. No. X649 dilution 1:100, validation: https://www.emfret.com/fileadmin/user_upload/Datasheets/X649.pdf
alpha-SMA (1A4), ThermoFisher, Cat. No. # 14-9760-82 dilution 1:100, validation: https://www.thermofisher.com/antibody/product/Alpha-Smooth-Muscle-Actin-Antibody-clone-1A4-Monoclonal/14-9760-82
hIL4-R (MAB230), R&D, Cat. No. MAB230-SP dilution 1:100, validation: https://www.rndsystems.com/products/human-il-4ralpha-antibody-25463_mab230
p34-Arc/ArpC2 (polyclonal), Merck , Cat. No. 07-227-I dilution 1:100, validation: https://www.merckmillipore.com/DE/de/product/Anti-p34-Arc-ARPC2,MM_NF-07-227-I-100UG
Arp2 (polyclonal), ECM Biosciences, Cat. No. AP3861 dilution 1:100, validation: https://ecmbio.com/collections/antibodies/products/ap3861
Arp3 (clone FMS338), Sigma, Cat. No. A5979 dilution 1:100, validation: https://www.sigmaaldrich.com/catalog/product/sigma/a5979
GPVI (clone HY101), Sigma, Cat. No. 14-9813-81 dilution 1:100, validation: https://www.thermofisher.com/antibody/product/GP6-Antibody-clone-HY101-Monoclonal/14-9813-81

anti-human
CD41 (clone HIP8), Biolegend, Cat. No. 303702 dilution 1:100, validation: https://www.biolegend.com/en-us/products/purified-anti-human-cd41-antibody-739
activated CD41 (clone PAC-1), Biolegend, Cat. No. 362802 dilution 1:100, validation: https://www.biolegend.com/en-us/products/purified-anti-human-cd41-cd61-antibody-9982
CD62P (clone AC1.2), Biolegend, Cat. No. 304906 dilution 1:100, validation: https://www.biolegend.com/en-us/products/pe-anti-human-cd62p-p-selectin-antibody-595
CD42b (clone HIP1), Biolegend, Cat. No. 303902 dilution 1:100, validation: https://www.biolegend.com/en-us/products/purified-anti-human-cd42b-antibody-733
Arp2/3 (clone 13C9), Merck, Cat. No. MABT95 dilution 1:100, validation: https://www.merckmillipore.com/DE/de/product/Anti-Arp2-3-complex-Antibody-clone-13C9,MM_NF-MABT95
phospho-myosin light chain 2 (pMLC, Thr18/Ser19), Cell Signaling, Cat. No. 3671S dilution 1:100, validation: https://www.cellsignal.de/products/primary-antibodies/phospho-myosin-light-chain-2-ser19-antibody/3671
fibrinogen (sheep polyclonal), Biorad, Cat. No. 4440-8004 dilution 1:100, validation: https://www.bio-rad.com/countrySelector?redirectUrl=https://www.bio-rad-antibodies.com/polyclonal/human-fibrinogen-antibody-4440-8004.html
Staphylococcus Aureus Antibody (polyclonal), Sigma, Cat. No. STAPH 11-248.2 dilution 1:100, validation: https://www.sigmaaldrich.com/catalog/product/mm/mab930

Secondary antibodies
goat anti-rabbit (polyclonal), Sigma, Cat. No. # A-11008 dilution 1:200, validation: https://www.thermofisher.com/antibody/product/Goat-anti-Rabbit-IgG-H-L-Cross-Adsorbed-Secondary-Antibody-Polyclonal/A-11008
donkey anti sheep (polyclonal), Sigma, Cat. No. SAB4600178-50UL, dilution 1:200, validation: https://www.sigmaaldrich.com/catalog/product/sigma/sab4600178
rat anti-mouse (polyclonal), Sigma, Cat. No. # 11-4011-85 dilution 1:200, validation: https://www.thermofisher.com/antibody/product/Rat-anti-Mouse-IgG-H-L-Secondary-Antibody-Polyclonal/11-4011-85

Depletion antibodies
purified rat anti-mouse GP1balpha antibodies and control immunoglobins, emfret, Cat. No. # R300 and #C301, 2µg/g mouse, validation: https://www.emfret.com/fileadmin/user_upload/Datasheets/R300.pdf
validation: https://www.emfret.com/fileadmin/user_upload/Datasheets/C301.pdf

Anti-Ly6G (clone 1A8), and control Rat IgG2a kappa(RTK2758), Biolegend, Cat. No. 127602 and Cat. No. 400501 100µg/mouse, validation: https://www.biolegend.com/fr-fr/search-results/purified-anti-mouse-ly-6g-antibody-4767, validation: https://www.biolegend.com/fr-fr/products/purified-rat-igg2a--kappa-isotype-ctrl-1845

Validation

see above

Animals and other organisms

Policy information about studies involving animals; ARRIVE guidelines recommended for reporting animal research

Laboratory animals	6-12 weeks-old female or male mice were used from the following mouse strains: C57BL/6, LifeAct-eGFP, PF4cre-Rosa26-Confetti fl/fl, IL4R/GPIb-tg, PF4cre-Arpc2 fl/fl, WASp-/-; PF4-Cre; Cyfip1fl/fl. Control and experimental groups were age- and sex-matched for all experiments. All mice live in standardized conditions where temperature, humidity, and hours of light and darkness are maintained at a constant level all year round.
Wild animals	No wild animals were included in this study.
Field-collected samples	No field-collected samples were included in this study.
Ethics oversight	All animal experiments were performed in compliance with all relevant ethical regulations for studies involving mice and were approved by the local legislation on protection of animals (Regierung von Oberbayern, Munich).

Note that full information on the approval of the study protocol must also be provided in the manuscript.

Human research participants

Policy information about studies involving human research participants

Population characteristics	Human blood was drawn from male and female healthy voluntary donors at the age of 25-40 years after informed consent was obtained from all subjects.
Recruitment	Both genders were equally represented in all our analyses.
Ethics oversight	Experiments involving human subjects have been approved by the ethical review board (LMU Munich) and complied with relevant regulation for experiments involving human samples.

Note that full information on the approval of the study protocol must also be provided in the manuscript.

Flow Cytometry

Plots

Confirm that:

- [x] The axis labels state the marker and fluorochrome used (e.g. CD4-FITC).
- [x] The axis scales are clearly visible. Include numbers along axes only for bottom left plot of group (a 'group' is an analysis of identical markers).
- [x] All plots are contour plots with outliers or pseudocolor plots.
- [x] A numerical value for number of cells or percentage (with statistics) is provided.

Methodology

Sample preparation	Blood: Whole Blood was incubated with antibodies for 20 minutes, and then diluted with FACS Lysing Buffer for flow cytometric analysis.<br>BAL: BAL was obtained by flushing the lung twice with 1ml PBS containing 2mM EDTA. Then, BAL was diluted 1:10 and incubated with Antibodies 20 min in dark.<br>Washed platelets: Platelets were isolated as described in the methods section; briefly, two centrifugation steps were performed (First: 70G, 20-35min, Second 1200G, 10 min) and cells were resuspended in Tyrode's Buffer. Then, cell staining and/or activation were performed in the presence of antibodies.
Instrument	BD Fortessa
Software	FACS Diva (BD) and FlowJo (BD)
Cell population abundance	Cell sorting was not performed
Gating strategy	For washed platelets no gating was performed. BAL and Blood were gated for singlets (FSC-A FSC-H). For BAL samples, a CD45+ TER119- cells were defined as leukocytes, TER119+ CD45- cells as Erythrocytes and Rainbow-Beads were detected as a separate autofluorescent population.

- [x] Tick this box to confirm that a figure exemplifying the gating strategy is provided in the Supplementary Information.
