# Supplementary material for: Vascular surveillance by haptotactic blood platelets in inflammation and infection
Source: Nat Commun. 2020 Nov 13;11:5778. doi: 10.1038/s41467-020-19515-0 (PMC7666582; doi:10.1038/s41467-020-19515-0)
Supplement: Supplementary file 1 — Supplementary Information [file 41467_2020_19515_MOESM1_ESM.pdf]

Supplementary Information for

Vascular surveillance by haptotactic blood platelets in inflammation and infection

Nicolai et al.

Supplementary Figure 1

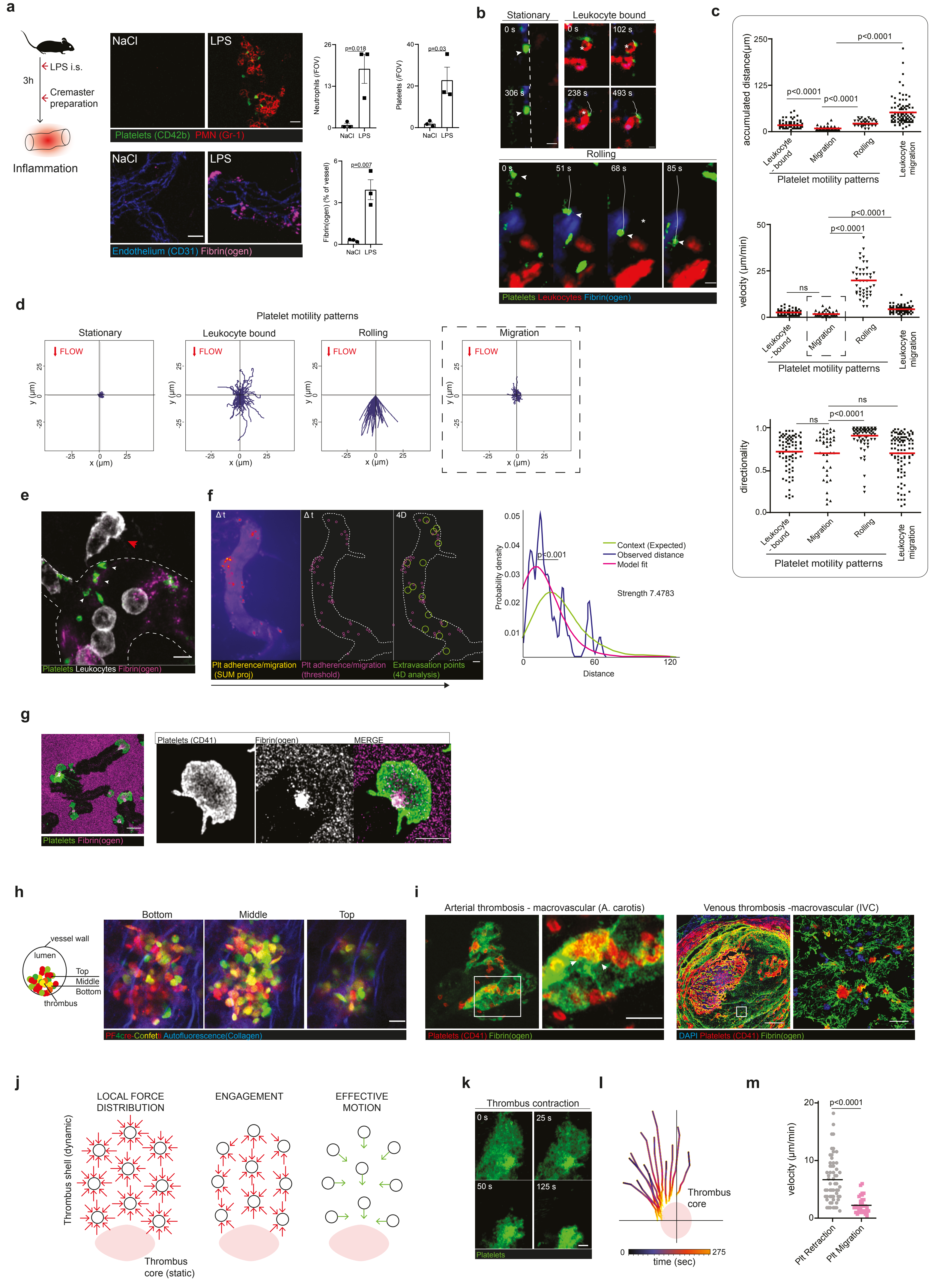

Supplementary Figure 1: Platelet morphology and motility patterns in vivo.

a-d, platelet motility patterns in the inflamed microvasculature showing (a) (left) cartoon of experimental setup, (middle) LPS induced cremaster inflammation model compared to NaCl control treatment, representative micrographs showing platelet, PMN, and fibrinogen recruitment/deposition in NaCl/LPS treated mice. (right) quantification of platelet, PMN, and fibrinogen recruitment/deposition, n=3 mice per group, ttest, scale bar= 20 µm. (b) exemplary observed motility patterns and (c) platelets analyzed for accumulated distance, velocity and directionality. Observed leukocyte migration is included for comparison. Data from 4 mice with n=47(adherent), n=81(leukocyte bound), n=39 (migrating, data drawn from Fig. 1b), n=44(rolling), and n=96 (leukocyte migration) in 5 mice. (d) x-y plots of platelet motility patterns. (c) Kruskal-Wallis, Dunn's post-hoc test. scale bar=5µm. Boxed areas are enlarged in Fig. 1b. **e-f**, platelet neutrophil colocalization in inflammation. (e) cremaster whole mount, visualization of adherent platelets (white arrows), deposited fibrinogen and neutrophils, red arrow: extravasated neutrophil. (f) colocalization analysis of platelets and neutrophil extravasation sites in the inflamed cremaster microvasculature. (left) image processing work-flow and (right) representative colocalization plot (n=3). Monte Carlo simulation, scale bar=10µm. **g,** platelet spreading and migration on fibrinogen surfaces in vitro, (left) overview and (right) representative lamellipodium-substrate interaction, scale bars= 5µm. **h,** representative micrographs of a FeCl-induced thrombus in PF4cre-Confetti mice. Top, middle and bottom views reveal no lamellipodia-forming platelets in the thrombus. scale bar=5µm. **i,** *in vivo* platelet recruitment and morphology in macrovascular arterial and venous thrombosis. (left) mouse carotid (arterial) and (right) vena cava (venous) thrombi, left: scale bar = 5µm, right: scale bar=200µm, inset: scale bar=20µm. **j,** simplified model of thrombus contraction based on a theoretical homogenous force field generated by recruited platelets. **k-m,** thrombus contraction in the mesenteric vasculature. (k) representative micrographs, (l) time-coded tracks and (m) velocity of *in vivo* retracting cells (mesenteric thrombus formation, n=59 cells in 3 mice) compared to migrating cells (data drawn from Fig. 1b), Mann-Whitney, scale bar = 10µm. All statistical tests are two-sided. Source data are provided as a Source Data file.

Supplementary Figure 2

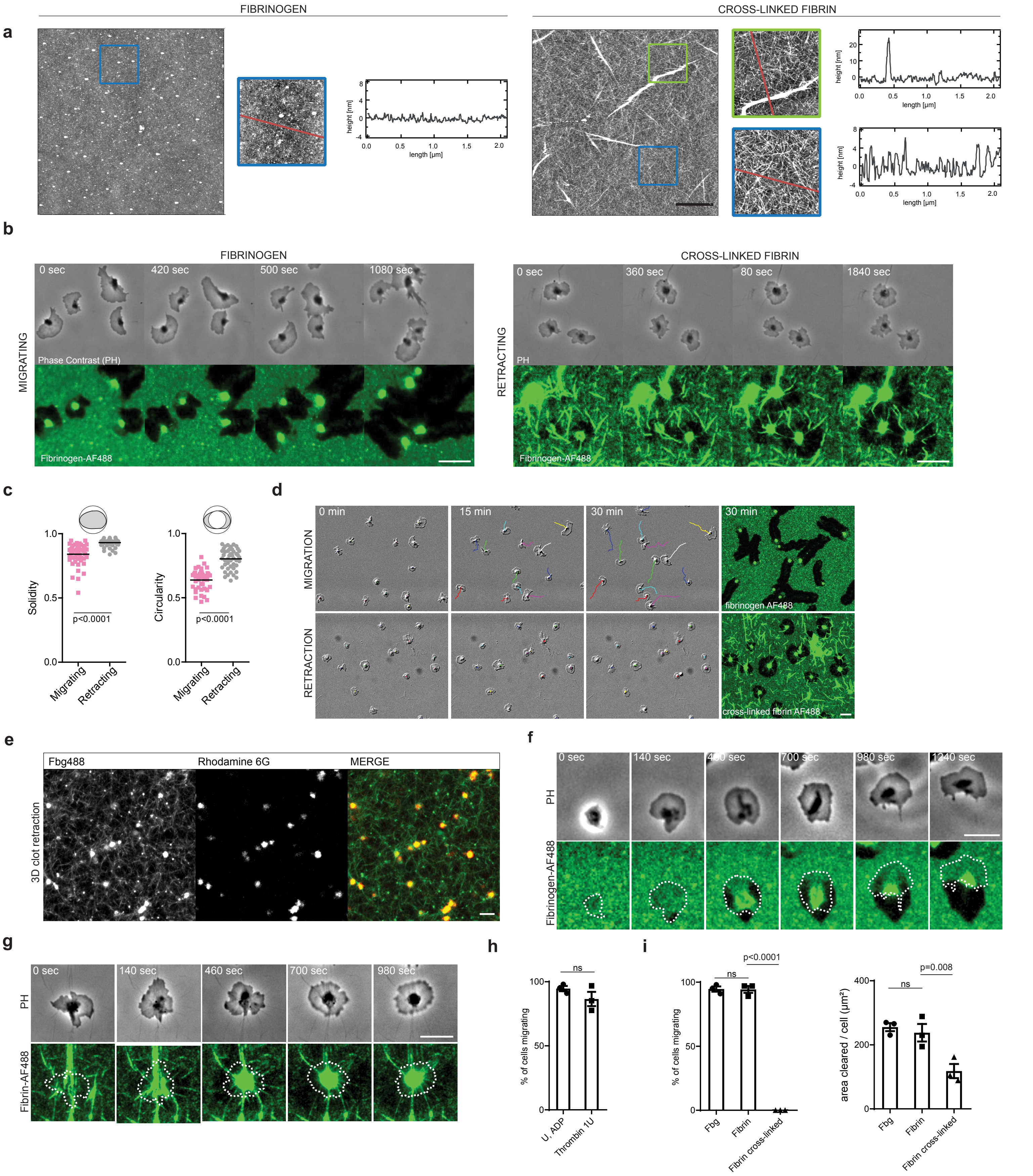

Supplementary Figure 2: Surface characteristics define platelet behavior *in vitro*

a, surface topology of fibrinogen and cross-linked fibrin substrates by Atomic Force Microscopy (AFM). (left) overview scan, (right) detailed scans and nanoscale line height profiles (indicated in red). scale bar=2µm. **b,c,** *in vitro* platelet morphology and behavior on fibrinogen and cross-linked fibrin surfaces. (b) micrographs over time and (c) comparison of shape descriptors solidity and circularity (migrating (n=43) and retracting (n=47) cells). Data from 3 experiments, Mann-Whitney. d, Difference interference contrast (DIC) of migrating (fibrinogen surface) and retracting cells (cross-linked fibrin surface). Fluorescent images depicting platelet traces after 30min. **e,** platelet morphology and substrate reorganization of 3D fibrin networks in an *in vitro* clot retraction assay. **f,g,** single cell morphology and substrate interaction on (f) fibrinogen and (g) cross-linked fibrin. h, percentage of migrating cells upon thrombin stimulation compared to ADP and thromboxane analog U46619 on fibrinogen coated surfaces (n=3), ttest. **i,** percentage of migrating platelets and cleared area per cell on fibrinogen, monomeric fibrin and cross-linked fibrin. n=3 experiments, ANOVA and Tukey post-hoc test. scale bars=5µm. error bars= s.e.m. All statistical tests are two-sided. Source data are provided as a Source Data file.

Supplementary Figure 3

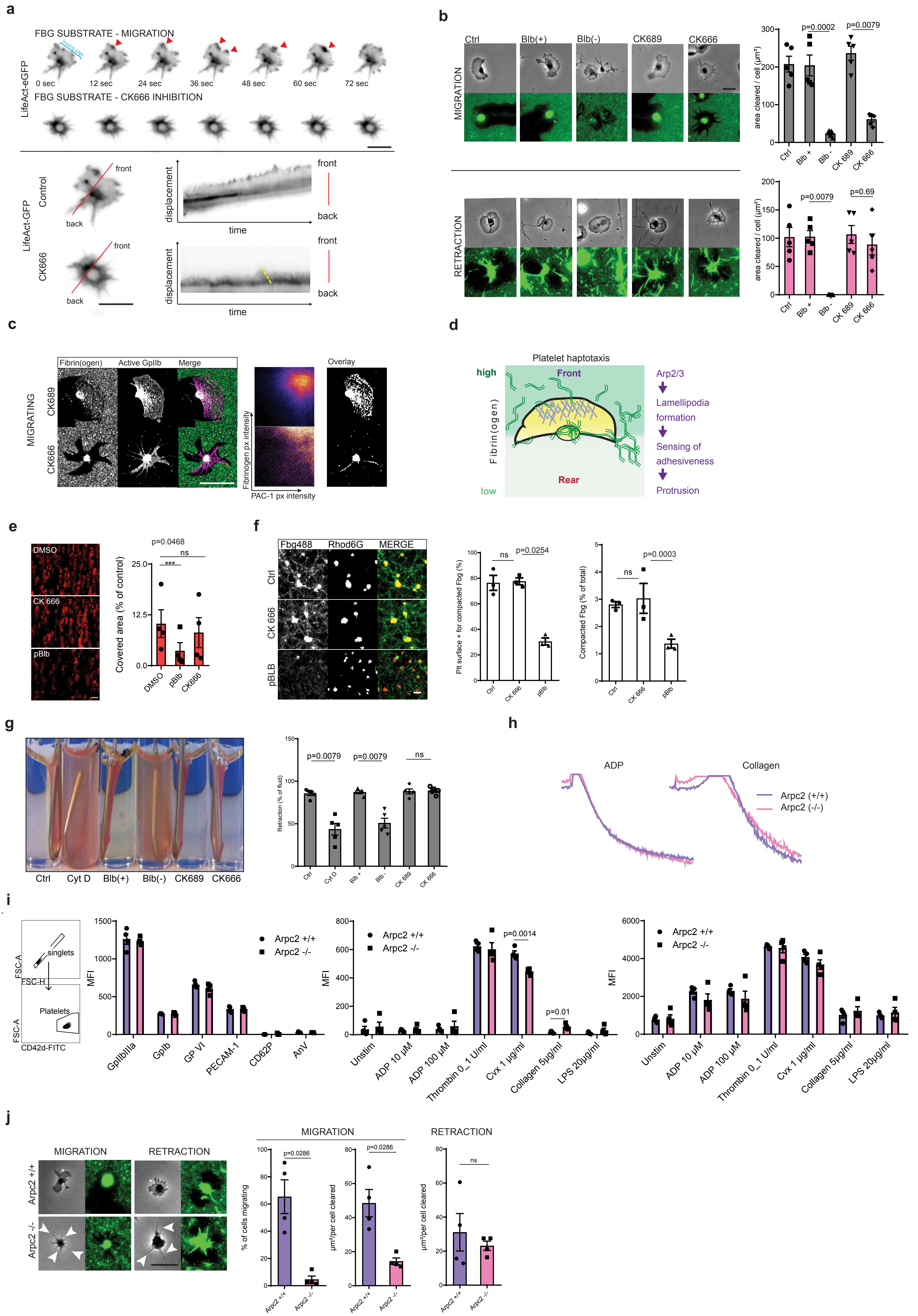

Supplementary Figure 3: Arp2/3 dependent cellular dynamics in migration and retraction

a, Actin dynamics in murine *LifeAct-eGFP* platelets migrating on fibrinogen in the presence and absence of CK666. top: videomicroscopy images, Red arrows: actin nucleation waves. bottom: representative kymographs of control and CK666 treated murine LifeAct-GFP platelets (red lines). CK666 treated platelets are immotile but show retrograde actin flow (yellow dashed lines), suggesting impaired adhesive coupling to the substrate. scale bars=5µm. **b,** effect of Arp2/3 inhibition with CK666 and myosin IIa inhibition with Blb(-) on migrating and retracting human platelets compared to CK689 and Blb(+) controls, (left) micrographs of representative platelets, (right) quantification of substrate removal, n=5 experiments, Mann-Whitney. Myosin inhibition was included as positive control globally abrogating platelet contractility. scale bar=5µm **c,** substrate engagement by active aIIbß3 integrin in representative control (CK689) and ARP2/3 inhibited (CK666) platelets on fibrinogen. (left) Fibrin(ogen) and active conformation of aIIbß3 (PAC-1) distribution, (middle) pixel intensity plots and (right) thresholded signal overlay micrographs. scale bar=5µm **d,** Platelets employ Arp2/3-dependent lamellipodia to sense substrate adesivness. Platelet lamellipodia protrude towards areas of higher adhesivness initiating haptotaxis of migrating platelets. **e,** *in vitro* flow chamber-based thrombosis model assessing human platelet deposition after treatment with DMSO (Control), para-Blebbistatin (pBlb) and CK666, n=4 experiments. Kruskal-Wallis, Dunns post-hoc test. scale bar=20µm. **f,** effect of myosin inhibition (pBlb) and Arp2/3 inhibition (CK666) on 3D clot retraction assay. left: maximum intensity projections of platelets and fibrin. right: compacted fibrin and platelets positive for compacted fibrin. ANOVA and Tukey post-hoc test, n=3. scale bar=5µm. **g,** clot retraction assay, representative micrographs after 30 min, and percentage of thrombus retraction measured by retrieved fluid, comparison of actin polymerization inhibitor Cytochalasin D (CytD) completely abrogating platelet shape change and control, myosin II inhibition (Blb-) and control (Blb+) and Arp2/3 inhibition (CK666) and control (CK689), n=5 experiments, Mann Whitney. **h-j,** *in vitro* analysis of *PF4-Cre-/+; Arpc2fl/fl* platelets (h), aggregometry of murine *Arpc2-/-* and *Arpc2+/+* platelet rich plasma (PRP) upon ADP (20µM) and collagen (0.1mg/ml) stimulation. (i), left: gating strategy and surface marker expression under steady state, middle: CD62P (P-selectin) expression and right: integrin aIIbß3 activation (JonA binding) upon agonist stimulation of *Arpc2+/+* and *Arpc2-/-* platelets, n=4 mice, ttests. (j), morphology and behavior of isolated murine *Arpc2-/-* and *Arpc2+/+* platelets on fibrinogen (migration) and cross-linked fibrin (retraction), representative micrographs, asterisks: lamellipodia, arrows: filopodia, percentage migrating and substrate clearance on fibrinogen and substrate clearance on cross-linked fibrin. n=4 mice, Mann Whitney. scale bar=5µm. error bars= s.e.m. All statistical tests are two-sided. Source data are provided as a Source Data file.

Supplementary Figure 4

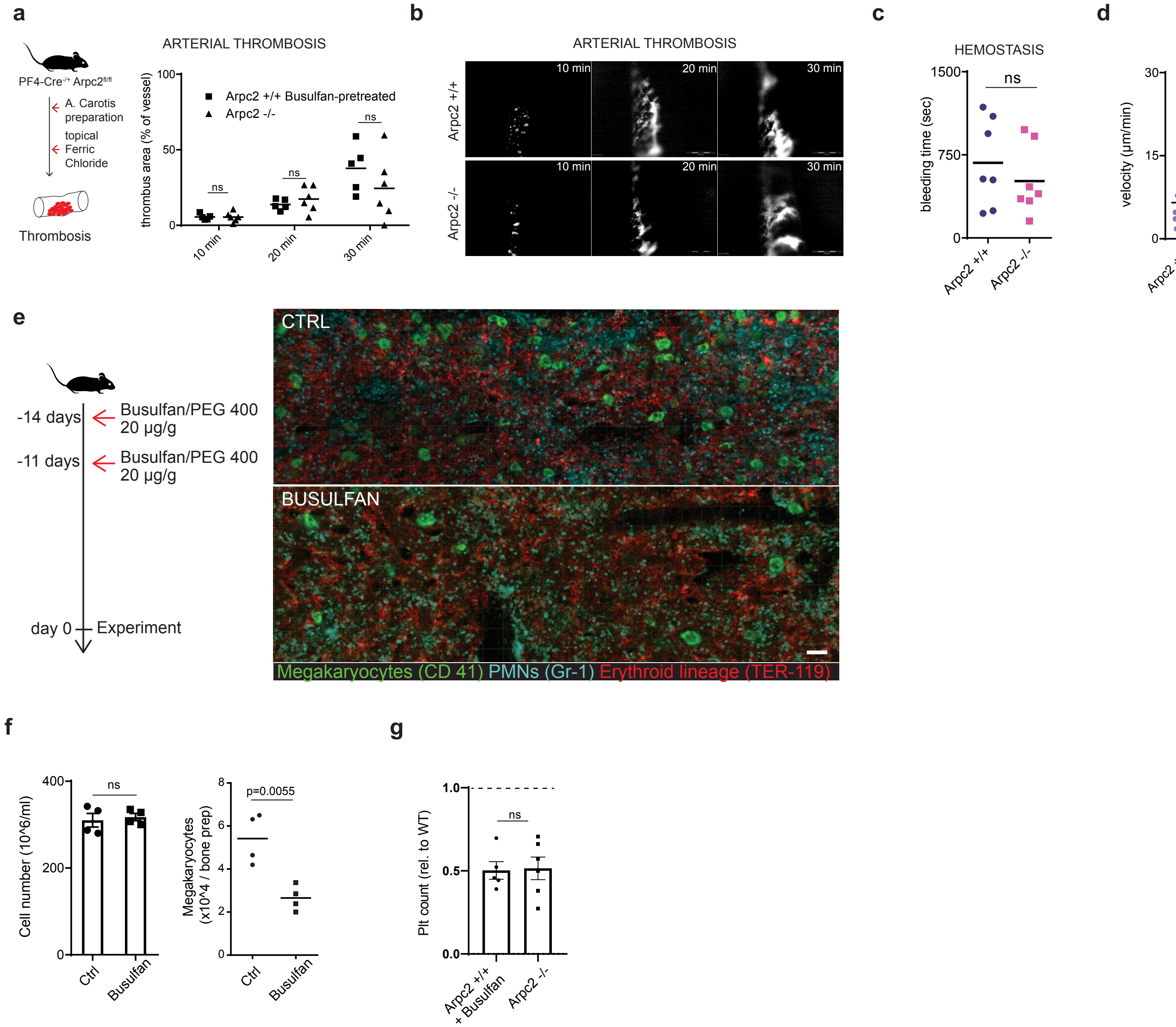

Supplementary Figure 4: Arp2/3 ablation *in vivo* does not affect hemostasis and thrombosis

a-d, effect of genetic Arpc2 deletion in murine platelets *in vivo* (a) carotid artery thrombus formation in Busulfan-treated animals +/+ (n=5 mice) and -/- (n=6 mice), b) carotid artery thrombus formation in *Arpc2+/+* and *Arpc2-/-* mice, Representative micrographs of thrombus formation over time. scale bars=100µm. (c), tail bleeding assay comparing *Arpc2+/+* and *-/-* animals, n=7. (d) *in vivo* clot retraction in the mesenteric vasculature of *PF4cre-Arpc2-/-* (n=85 cells in 3 mice) and WT mice (control drawn from Supplementary Fig. 1m). (a) Kruskall-Wallis, Dunn's post-hoc test, (c-d) ttest. scale bars=5 µm. **e-f,** analysis of bone marrow from Busulfan-treated and control WT mice. (e) experimental protocol and bone marrow whole mount depicting megakaryocytes, erythrocytes, and neutrophils/monocytes. scale bar=5µm. (f) bone marrow cellularity and flow cytometric quantification of CD41+CD42d+ megakaryocytes, n=4 mice, ttests. **g,** platelet counts in busulfan-depleted *Arpc2+/+* animals and *Arpc2-/-*, relative to controls, *Arpc2+/+* Busulfan n=5, *Arpc2-/-* n=6, ttest. error bars= s.e.m. All statistical tests are two-sided. Source data are provided as a Source Data file.

Supplementary Figure 5

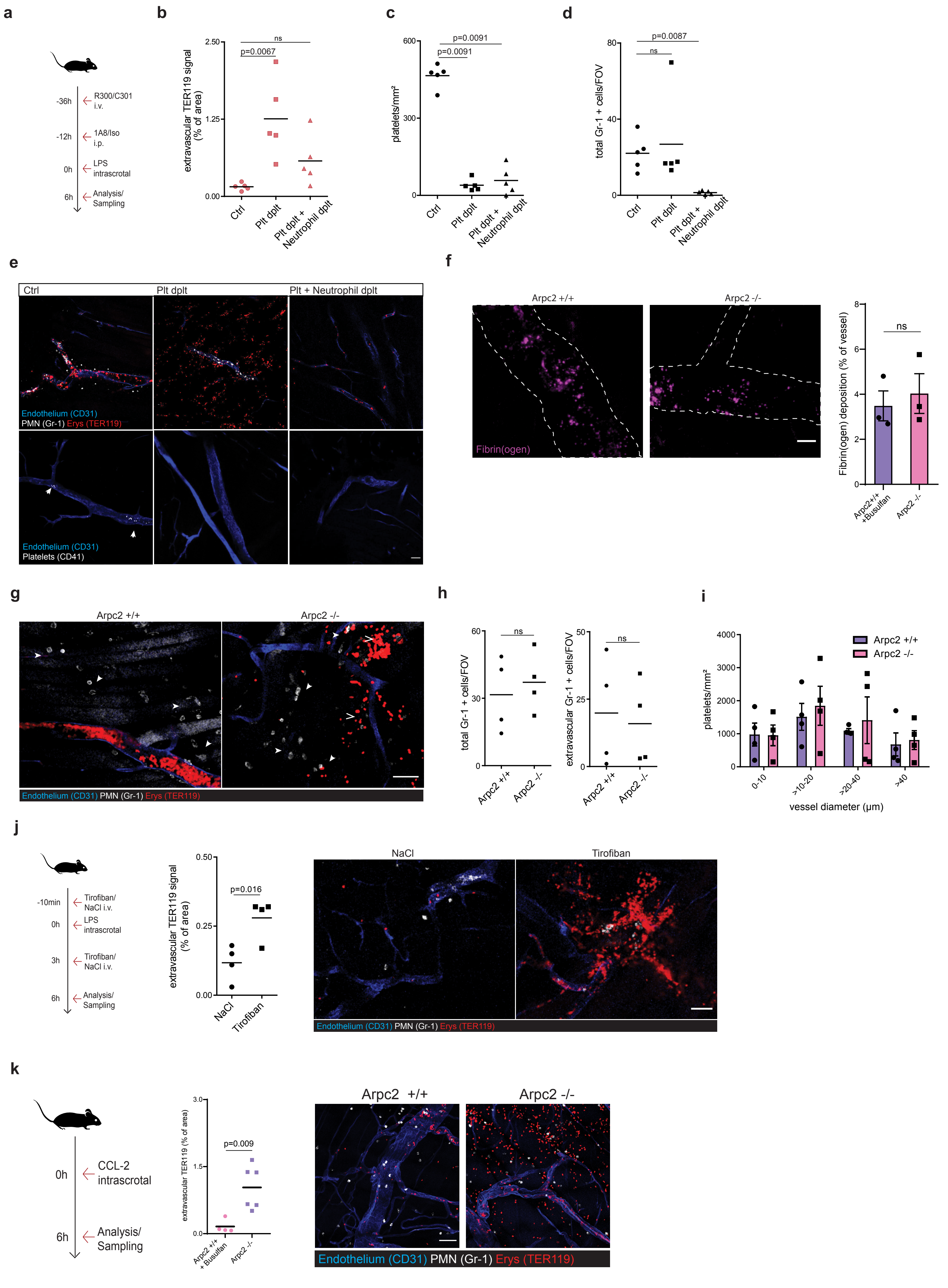

Supplementary Figure 5: Migrating platelets prevent microbleeds caused by extravasating neutrophils
a-e, effect of platelet and/or neutrophil depletion on inflammatory bleeding in LPS-induced inflammation in the cremaster muscle. (a) depletion protocol and (b) bleeding assessment by quantification of extravasated erythrocyte content. (c) quantification of platelet and (d) monocyte/neutrophil (Gr-1+) recruitment to the inflamed cremaster vasculature. (e), representative cremaster whole mount stainings. white arrows: platelets. (b-d) n=5 mice, Kruskal-Wallis, Dunn's post-hoc test. scale bar=5μm. **f,** fibrin(ogen) deposition in *Arpc2 +/+* and *-/-* mice in LPS-induced inflammation, in the cremaster. Scale bar = 10 μm, ttest. **g-i,** inflamed cremaster microvasculature in *Arpc2+/+* and *-/-* mice (g) extravasated neutrophils/ monocytes (Gr-1+, white arrows) and erythrocytes (TER119+, open arrows). (h), neutrophil/ monocyte recruitment and extravasation and (i) platelet recruitment to the inflamed cremaster in *Arpc2+/+* and *-/-* mice, n=4 mice, ttest. scale bar=50μm. **j,** bleeding assessment in the inflamed cremaster microvasculature of Tirofiban and NaCl control treated mice, used treatment protocol and quantification of extravascular TER119 signal (n=4), ttest. Scale bar=50μm. **k,** bleeding assessment in the inflamed cremaster microvasculature of *Arpc2+/+* and *-/-* mice, challenged with CCL-2, representative whole mount stains stained for erythrocytes (TER119) and vasculature (CD31) and quantification of extravascular TER119 signal (*Arpc2+/+*: n=4, *Arpc2-/-*; n=6 mice per group), ttest. Scale bar=50μm. error bars= s.e.m. All statistical tests are two-sided.Source data are provided as a Source Data file.

Supplementary Figure 6

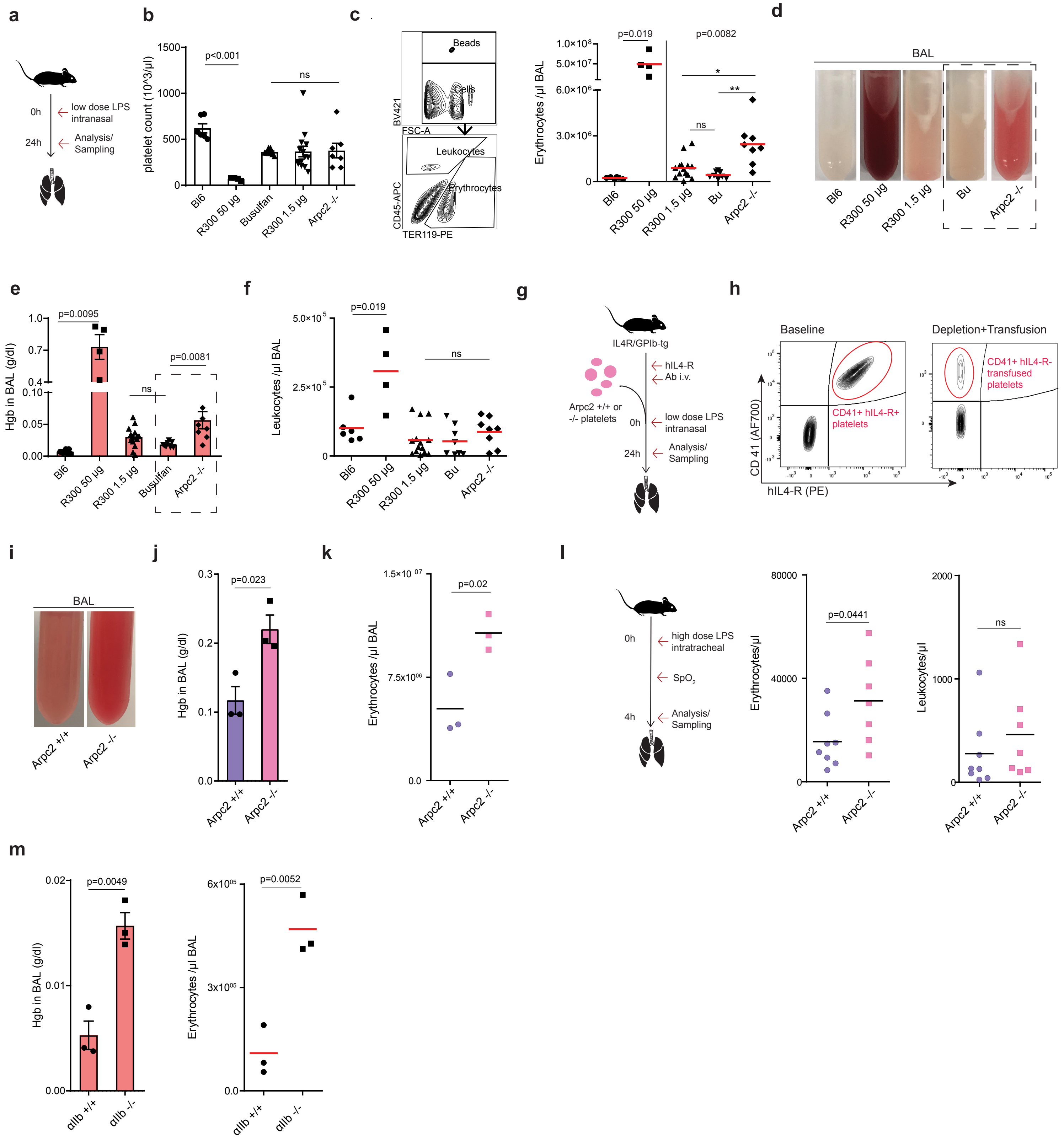

Supplementary Figure 6: Platelet spreading and migration contributes to vascular integrity in acute lung injury **a-f,** (sub-)acute lung injury model. (a), setup of model, (b-f), comparison of *Arpc2-/-* bleeding with untreated, antibody mediated platelet deletion/reduction (R300) and chemical megakaryocyte targeted platelet reduction (Busulfan) in WT (*Arpc2+/+*) mice in (sub-) acute lung injury (ALI). (b), peripheral platelet counts, (c), right: gating strategy for erythrocytes and leukocytes in BAL as shown in Suppl. Fig. 6c,f,k,l-m, left:erythrocytes (TER119+) in BAL, (d) representative photographs of bronchoalveolar lavage (BAL), (e) hemoglobin content in BAL and (f) leukocyte content in BAL. (b-f) Bl6: n= 6, R300 50µg: n=4; Mann-Whitney; R300 1,5µg: n=14, Busulfan: n=8, *Arpc2-/-*:n=7 mice per group; Kruskall-Wallis, Dunn's post-hoc test. Boxed areas are enlarged in Fig. 5c-d. **g-k,** adoptive transfer experiment. (g) setup and (h) gating strategy and flow cytometric analysis of platelets at baseline and after depletion/transfusion of *Arpc2 -/-* and control platelets in *hIL4R/GPIb-tg* mice. (i) exemplary photograph and (j) Hemoglobin and (k) erythrocyte content in BAL. n=3 mice, ttest. **l,** hyperacute lung injury model, supplement to Fig. 5g,h. erythrocyte and leukocytes content in BAL, *Arpc2+/+*: n=8, *Arpc2-/-*: n=7 mice per group, ttest. **m,** acute lung injury model of *αIIb +/+* and *-/-* bone marrow chimeric mice, hemoglobin and erythrocyte content in BAL of n=3 mice, ttest. error bars= s.e.m. All statistical tests are two-sided. Source data are provided as a Source Data file.

Supplementary Figure 7

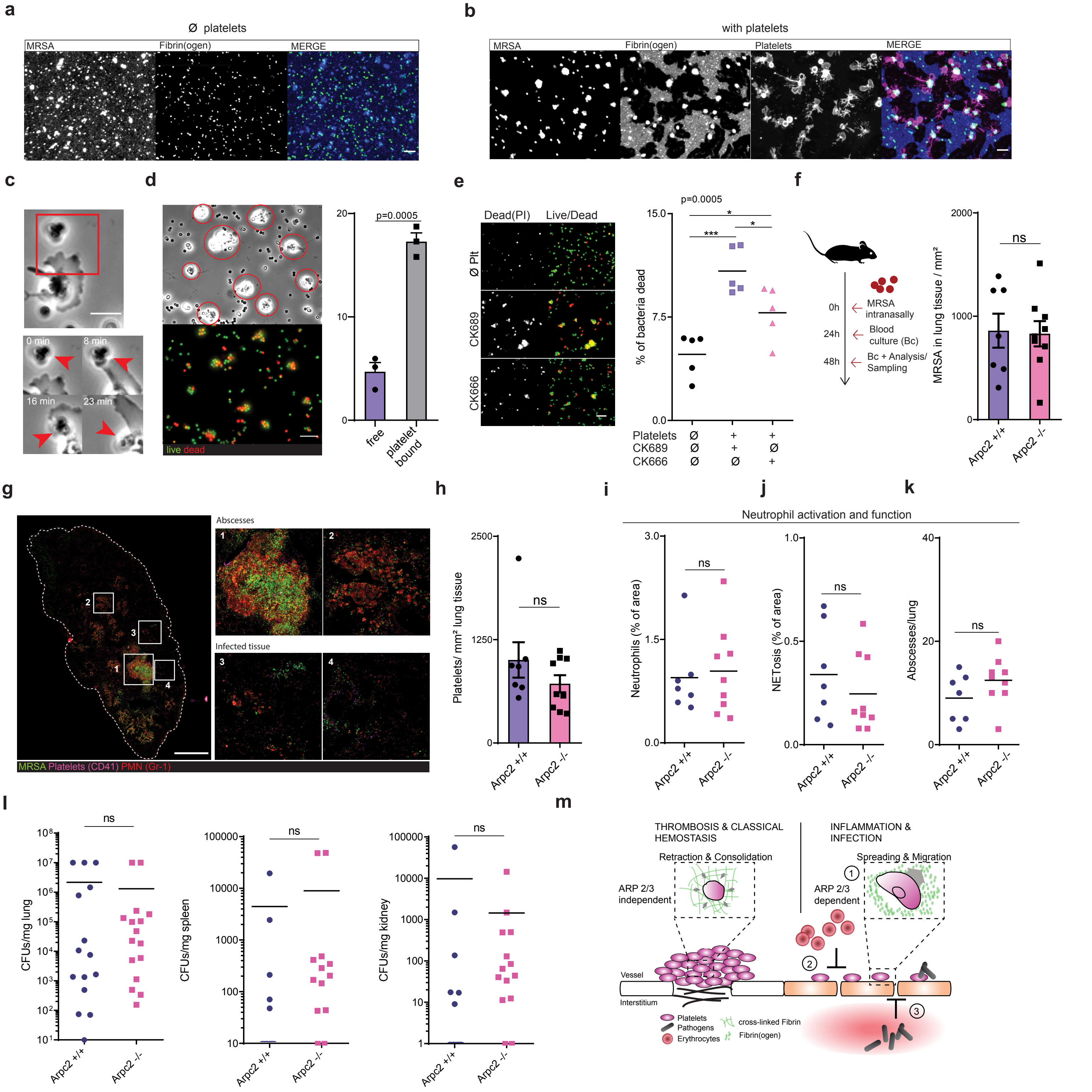

Supplementary Figure 7: Platelet interaction with MRSA *in vitro* and *in vivo*

a,b MRSA biofilms *in vitro* (a) without and (b) after addition of platelets. scale bar=10µm. **c,** MRSA uptake by migrating platelets. scale bar =5µm **d,** comparison of live/dead stain (propidium iodide (PI)/Sytox Green) of free bacteria and bacteria collected and bundled by platelets. n=3 experiments, ttest. **e,** Live/Dead stain (propidium iodide(PI)/Sytox Green) of MRSA biofilms without platelets and with CK689 or CK 666 treated platelets, n=5 experiments, ANOVA and Tukey's post-hoc test. Scale bar=10µm. **f-l,** MRSA lung infection model of *Arpc2*+/+ and -/- bone marrow chimeric mice, supplement to Fig. 7c-h. (f) MRSA content in the lung. (g), lung of *Arpc2*+/+ animal 48h p.i. comprised of MRSA abscesses and non-abscessing infected lung tissue with disseminated MRSA. scale bar=10mm (h) platelet recruitment, (i), count of MRSA lung abscesses/mouse, (j), overall neutrophil and (k), citH3 staining indicative for NETosis and NETosis-primed neutrophils in infected lungs of *Arpc2*+/+ and -/- mice. (l), lung CFUs in *Arpc2*+/+ and -/- mice. (f-k) Mann-Whitney, *Arpc2*+/+: n=7, *Arpc2*-/-: n=9 mice per group (l), *Arpc2*+/+: n=15, *Arpc2*-/-: n=16 mice per group, Mann-Whitney. **m,** graphical abstract: Haptotactic platelets secure vascular borders and fight bacterial invasion. Platelets mechanically probe their microenvironment by pulling on adhesive ligands such as fibrin fibers. This leads to Arp2/3-independent retraction and consolidation of 3D clots in thrombosis and classical hemostasis. In contrast, intravascular deposition of thin layers of fibrin(ogen) during inflammation and infection triggers Arp2/3-dependent platelet spreading and haptotactic migration (1). Arp2/3-dependent processes prevent inflammatory bleedings (2) and defend the vasculature against invading pathogens (3). error bars= s.e.m. All statistical tests are two-sided. Source data are provided as a Source Data file.
